# Supplementary material for: Impact of Iron Oxide on Anaerobic Digestion of Frass in Biogas and Methanogenic Archaeal Communities’ Analysis
Source: Biology (Basel). 2024 Jul 17;13(7):536. doi: 10.3390/biology13070536 (PMC11273746; doi:10.3390/biology13070536)
Supplement: Supplementary file 1 [file biology-13-00536-s001.zip › biology-3069705-supplementary.pdf]

Supplementary Material

1 Supplementary Figures and Tables

1.1 Supplementary Figures

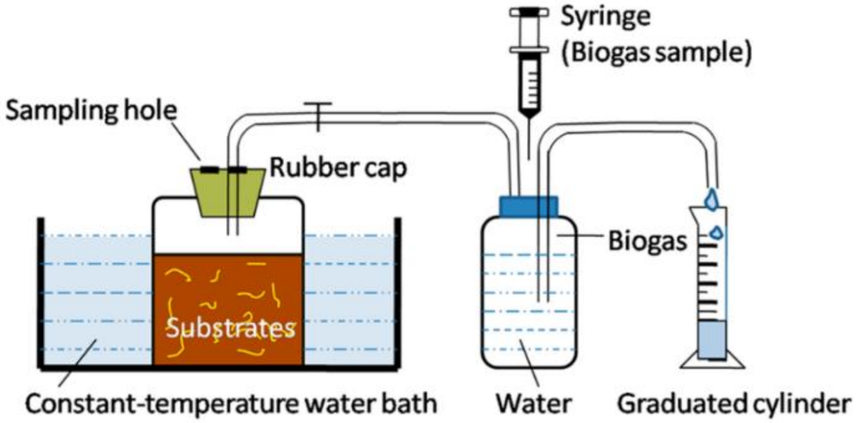

Figure S1. Schematic view of the AD apparatus.

1.2 Supplementary Tables

Table S1. Fermentation characteristics of raw materials

Parameter	TS (/%)	VS (/%)	C (/%)	N (/%)	H (/%)	S (/%)	C/N	C/H
Frass	71.78	52.32	29.85	2.96	5.14	0.84	10.10	5.80
Rice straw	93.57	64.71	34.5	1.09	4.86	0.062	31.61	7.10
